# Host and Parasite Transcriptomic Changes upon Successive Plasmodium falciparum Infections in Early Childhood

**DOI:** 10.1128/mSystems.00116-20

**Published:** 2020-07-07

**Authors:** Katie R. Bradwell, Drissa Coulibaly, Abdoulaye K. Koné, Matthew B. Laurens, Ahmadou Dembélé, Youssouf Tolo, Karim Traoré, Amadou Niangaly, Andrea A. Berry, Bourema Kouriba, Christopher V. Plowe, Ogobara K. Doumbo, Kirsten E. Lyke, Shannon Takala-Harrison, Mahamadou A. Thera, Mark A. Travassos, David Serre

**Affiliations:** a Institute for Genome Sciences, University of Maryland School of Medicine, Baltimore, Maryland, USA; b Malaria Research and Training Center, University of Science, Techniques and Technologies, Bamako, Mali; c Malaria Research Program, Center for Vaccine Development and Global Health, University of Maryland School of Medicine, Baltimore, Maryland, USA; d Duke Global Health Institute, Duke University, Durham, North Carolina, USA; Oxford Nanopore Technologies

**Keywords:** malaria, transcriptomics

## Abstract

Children are highly susceptible to clinical malaria, and in regions where malaria is endemic, their immune systems must face successive encounters with Plasmodium falciparum parasites before they develop immunity, first against severe disease and later against uncomplicated malaria. Understanding cellular and molecular interactions between host and parasites during an infection could provide insights into the processes underlying this gradual acquisition of immunity, as well as to how parasites adapt to infect hosts that are successively more malaria experienced. Here, we describe methods to analyze the host and parasite gene expression profiles generated simultaneously from blood samples collected from five consecutive symptomatic P. falciparum infections in three Malian children. We show that the data generated enable statistical assessment of the proportions of (i) each white blood cell subset and (ii) the parasite developmental stages, as well as investigations of host-parasite gene coexpression. We also use the sequences generated to analyze allelic variations in transcribed regions and determine the complexity of each infection. While limited by the modest sample size, our analyses suggest that host gene expression profiles primarily clustered by individual, while the parasite gene expression profiles seemed to differentiate early from late infections. Overall, this study provides a solid framework to examine the mechanisms underlying acquisition of immunity to malaria infections using whole-blood transcriptome sequencing (RNA-seq).

**IMPORTANCE** We show that dual RNA-seq from patient blood samples allows characterization of host/parasite interactions during malaria infections and can provide a solid framework to study the acquisition of antimalarial immunity, as well as the adaptations of P. falciparum to malaria-experienced hosts.

## Abstract (precis)

We show that dual RNA-seq from patient blood samples allows characterization of host/parasite interactions during malaria infections and can provide a solid framework to study the acquisition of antimalarial immunity, as well as the adaptations of P. falciparum to malaria-experienced hosts.

## INTRODUCTION

Despite tremendous progress in the last decades, malaria still has devastating consequences throughout Africa, where Plasmodium falciparum causes more than 200 million malaria cases and close to half a million deaths every year, the majority of them children (1). In Mali, malaria remains a leading cause of death in children under 5 years of age (2). In Bandiagara, a town of approximately 14,000 inhabitants in central Mali, malaria is highly seasonal with a transmission that peaks in September (3) and each child typically experiences one or two clinical episodes of malaria every year (4).

With repeated exposures to malaria parasites, children living in high-transmission settings gradually acquire immunity against the disease, first against severe malaria manifestations, then against milder symptoms that characterize uncomplicated malaria, until they eventually develop asymptomatic infections (5) with increased parasite clearance (6, 7). A better understanding of the processes accompanying the acquisition of immunity could shed light on the mechanisms underlying disease resistance and parasite tolerance and could guide more effective antimalarial treatments. Antidisease immunity is thought to be partially mediated by recognition of multiple antigens, and longitudinal studies following children from infancy to adulthood have demonstrated recognition of an increasing number of antigens and antigen variants over time (8). Innate immunity may also play a role in protecting against malaria and in modulating the adaptive immune response afforded by this repertoire (9). However, adaptive immunity appears to be relatively short-lived, as evidenced by loss of immunity upon discontinued exposure (10). Aside from “strain-specific” acquired immunity, the concept of a “strain-transcending” immunity that may be mediated by host age has been proposed (11), although it is important to note that severe malaria does occur in older children in low-transmission areas (12). Parasite tolerance, possibly acquired via immunoregulatory mechanisms (13), remains a poorly characterized yet intriguing avenue of research. Study of immune cell repertoires have shown that complicated malaria cases present different CD4^+^ T-cell phenotypes than those seen in uncomplicated or asymptomatically infected individuals (14). Platelets, which act as first responders to infection, secrete platelet factor 4 (which lyses the parasitic vacuole), and present antigens in the context of major histocompatibility complex (MHC) class I, have also been shown to exhibit clear differences in counts between patients with complicated malaria (greater thrombocytopenia) and those with uncomplicated malaria (7).

Gene expression analyses have the potential to complement immunological studies and could reveal molecular processes underlying the acquisition of immunity to malaria. Transcriptome sequencing (RNA-seq) studies have thus reported gene expression differences between malaria-naive and -experienced individuals (15), as well as between severe and uncomplicated malaria cases (16). In addition, microarray studies have shown that recent and heavy exposure to malaria is associated with a loss of proinflammatory cytokine production (17), and higher levels of the anti-inflammatory cytokine interleukin 10 (IL-10) (18).

To date, most malaria gene expression studies focused either on the host response to infection or on parasite gene expression and its association with disease phenotypes, with the exception of a few studies that characterized general interactions between infecting malaria parasites and their hosts (16, 19). Simultaneous characterization of host and parasite gene expression profiles, sometimes referred to as dual RNA-seq, could provide novel perspectives on the interactions between host and pathogen during an infection (20) and address an important but understudied aspect of immunity. In particular, study of the dynamic changes occurring over successive infections could identify molecular pathways involved in host immunity acquisition and mechanisms used by parasites to overcome the immunity of more experienced hosts. Here, we describe the parasite and host gene expression profiles generated from blood samples collected in the context of a malaria incidence study (4) from three young Malian children during five successive clinical malaria episodes. We show that RNA-seq provides robust characterization of both organisms’ transcriptomes without requiring sample processing or culture. We present statistical analyses of the temporal changes in gene expression and show that the gene expression profiles of both organisms cluster differently over time. We also demonstrate how RNA-seq data can support (i) gene expression deconvolution analyses to estimate the proportions of the different parasite stages and white blood cell (WBC) subsets in each sample, (ii) analysis of host-parasite gene coexpression, and (iii) robust genotyping to examine the complexity of each infection.

## RESULTS

### Changes in host and parasite gene expression over successive infections.

We simultaneously analyzed the parasite and host gene expression profiles from three Malian children (aged 1 to 2 years old) enrolled in a longitudinal study of malaria incidence in Bandiagara, Mali, between 2009 and 2014 (4). For each child, we extracted RNA from five blood samples collected during successive symptomatic P. falciparum infections for a total of 15 samples (for details, see Table S1 in the supplemental material). After ribosomal and globin RNA depletion, we prepared a stranded RNA-seq library and generated 34 to 67 million read pairs from each sample (Table S2).

10.1128/mSystems.00116-20.1 TABLE S1 Subject symptomology and infection dates. Download Table S1, XLSX file, 0.01 MB. Copyright © 2020 Bradwell et al. 2020 Bradwell et al. This content is distributed under the terms of the Creative Commons Attribution 4.0 International license.

10.1128/mSystems.00116-20.2 TABLE S2 Read mapping statistics. Download Table S2, XLSX file, 0.02 MB. Copyright © 2020 Bradwell et al. 2020 Bradwell et al. This content is distributed under the terms of the Creative Commons Attribution 4.0 International license.

To test if any of those infections contained more than one species of *Plasmodium* parasites (21), we mapped the reads to the genome sequences of different *Plasmodium* species infecting humans. In all samples, the reads mapping to P. ovale, P. malariae, and P. vivax represented less than 0.68% of all *Plasmodium* reads, suggesting that these blood samples were infected with only P. falciparum. Overall, 17 to 91% of the reads mapped to the human genome and 5 to 78% of the reads mapped to the P. falciparum genome (Table S2). After stringent quality filters, we obtained 5 to 24 and 1 to 12 million reads mapping to the human and P. falciparum genomes, respectively. The majority of reads mapped to annotated coding regions (∼80% for human and ∼97% for P. falciparum) and provided sufficient information to analyze the expression levels of 8,896 host and 2,822 parasite genes (see Materials and Methods).

**FIG 1:**
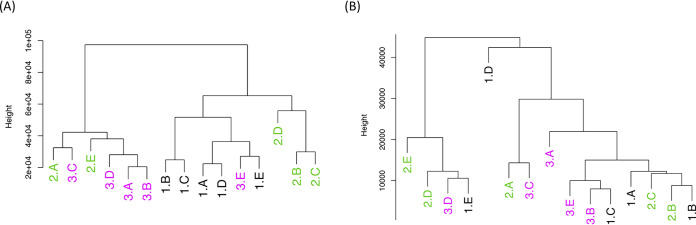
Unsupervised clustering of the 15 samples according to the host (A) and parasite (B) gene expression profiles. The colors and numbers (1 to 3) indicate which patient the sample is derived from. The letters distinguish the five symptomatic infections from each patient, with A representing the earliest infection and E the latest. Tree height refers to dissimilarities in terms of squared Euclidean distance between cluster means.

To assess how host and parasite gene expression profiles change over successive infections, we compared the transcriptomes of the 15 samples (3 children × 5 successive infections). Unsupervised clustering revealed that host gene expression profiles tended to cluster each child’s successive infections together (Fig. 1A), while the P. falciparum transcriptomes generated from the same successive infections tended to differentiate early from late infections, regardless of the individual (Fig. 1B). To further investigate this pattern, for each host and parasite gene, we tested whether the expression was influenced by the host and/or sequential infections (i.e., whether it was the first, second, third, fourth, or fifth infection) using a statistical framework that assessed whether the expression levels changed consistently over time. Consistent with the hierarchical clustering results, a greater number of host genes were differentially expressed according to the individual than the number of the infection (e.g., 4,581 versus 1,042 at a false discovery rate [FDR] of 0.2), while a greater number of parasite genes were differentially expressed according to the number of the infection rather than the individual (0 versus 68, FDR = 0.2) (Table 1 and Tables S3 and S4).

**TABLE 1:** Number of host and parasite genes differentially expressed according to the patient and the number of the infection^*a*^

Transcriptome	No. of genes tested	DE according to patient or infection no.	No. of DE genes at:	
			FDR = 0.2	FDR = 0.1
Host	8,896	Patient	4,581	2,876
		Infection no.	1,042	97
Parasite	2,822	Patient	0	0
		Infection no.	68	11

a Only genes expressed at more than 10 counts per million in more than six samples were tested (see Materials and Methods).

10.1128/mSystems.00116-20.3 TABLE S3 Host genes differentially expressed over successive infections and patients. Download Table S3, XLSX file, 2.8 MB. Copyright © 2020 Bradwell et al. 2020 Bradwell et al. This content is distributed under the terms of the Creative Commons Attribution 4.0 International license.

10.1128/mSystems.00116-20.4 TABLE S4 Parasite genes differentially expressed over successive infections and by patients. Download Table S4, XLSX file, 0.9 MB. Copyright © 2020 Bradwell et al. 2020 Bradwell et al. This content is distributed under the terms of the Creative Commons Attribution 4.0 International license.

**FIG 2:**
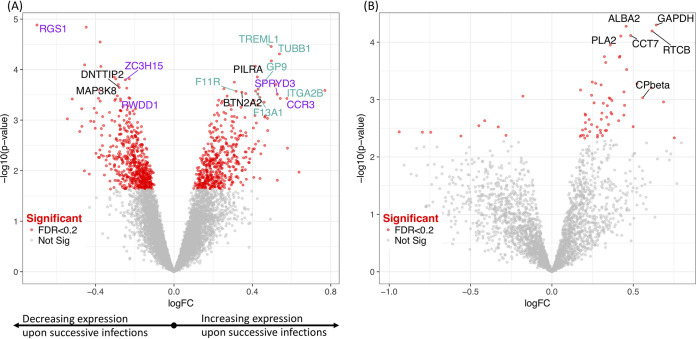
Volcano plot showing the results of the differential gene expression according to the number of successive infections for the host (A) and parasite (B) genes. Each dot represents one gene and is displayed according to the log fold change in expression (*x* axis) and the statistical significance of the association (*y* axis, in –log_10_ of the *P* value). Red dots indicate genes deemed to be differentially expressed (FDR = 0.2). Genes that increased in expression over the course of the five successive infections are shown by positive log fold change values, and those that decreased in expression are shown by negative log fold change values. Selected genes discussed in the text are labeled and, for the host, are color coded based on their functional annotation (immunoregulatory functions shown in black, platelet aggregation in turquoise, and G-protein signaling in purple).

**FIG 3:**
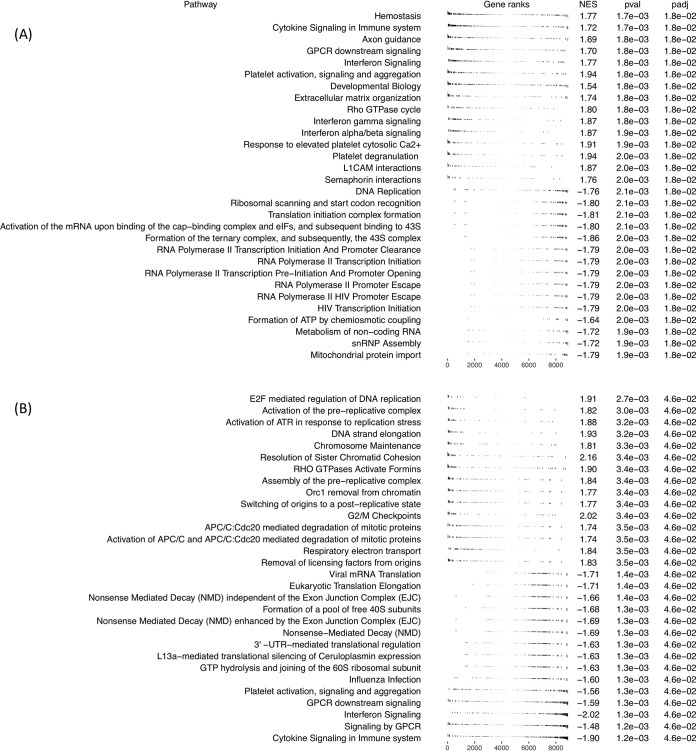
GSEA analysis of the human transcriptome by infection number (A) and patient 1 versus patient 2 (B). (A) The top 15 plots show the top 15 pathways upregulated over successive infection numbers, and the bottom 15 plots show the top 15 pathways downregulated over successive infection numbers. (B) The top 15 plots show the top 15 pathways upregulated in patient 2, and the bottom 15 plots show the top 15 pathways downregulated in patient 2.

Several of the host genes whose expression changed the most (and consistently) over successive infections were involved in G-protein signaling, platelet aggregation, and immunoregulation (Fig. 2A and Table S3). To systematically examine whether some pathways were disproportionally represented among the genes differentially expressed according to the number of the infection (*n* = 97, FDR = 0.1), we performed enrichment analyses. PANTHER overrepresentation test (22) suggested that blood coagulation was also influenced by the number of the infection (*P* value = 1.63 × 10^−5^). Gene set enrichment analysis (GSEA) for Reactome pathways (Fig. 3A) confirmed the roles of G-protein signaling and blood coagulation, as well as revealed enrichment in other pathways such as cytokine signaling. Table S5 shows the full results and information on the leading edge genes that drive the enrichment. Only a handful of parasite genes reached statistical significance (Fig. 2B and Table S4), including phospholipase A2, Alba 2, and glyceraldehyde-3-phosphate dehydrogenase, and no specific pathway was statistically enriched.

10.1128/mSystems.00116-20.5 TABLE S5 GSEA results for human transcriptomic changes by infection number and by patient 1 versus patient 2. Leading edge genes are provided for pathways enriched at adjusted *P* < 0.05. Download Table S5, XLSX file, 0.4 MB. Copyright © 2020 Bradwell et al. 2020 Bradwell et al. This content is distributed under the terms of the Creative Commons Attribution 4.0 International license.

The host genes differentially expressed among the three children (Table S3) included CD36, also known as glycoprotein IV, a membrane protein present on the surface of many cell types that facilitates the binding and activation of platelets and monocytes (23) and is hypothesized to influence the host response to P. falciparum infection (7). Despite the large number of differentially expressed genes (2,876 genes at a FDR = 0.1), PANTHER analysis did not reveal any significant enrichment after multiple testing correction. GSEA results produced statistically significant results (*P* < 0.05) only for the child 1 versus child 2 comparison (Fig. 3B and Table S5), and leading edge analysis did not place CD36 in any significantly enriched pathways. Child 1, the oldest of the three children, showed enrichment of platelet-related and cytokine-signaling related pathways compared to both of the other children (although child 1 versus child 3 did not reach significance), reflecting the findings of enrichment by infection number.

### Coexpression of host and parasite genes.

Joint characterization of host and parasite gene expression profiles from the same blood sample provides an opportunity to look for interactions, either directly between host and pathogen proteins, or indirectly as one molecular pathway in one organism may regulate a separate process in the other organism. We searched for putative interactions by measuring the correlation between the expression levels of each pair of host gene-parasite gene across all 15 infections. We identified 2,690 pairs with a Spearman’s coefficient of correlation *R*^2^ > 0.9 (see, e.g., Fig. S1 in the supplemental material). This high extent of correlation observed between host and parasite gene expression was much greater than one would expect solely by chance (*P* = 0.024, based on 500 permutations), and indeed, only 709 gene pairs should display such high correlations by chance (corresponding to a FDR of 0.26, see Materials and Methods). Thus, despite the small sample size of the current study, our analyses demonstrate that dual RNA-seq can identify statistically significant host/pathogen correlations at the transcript level and could provide a framework to rigorously assess interactions occurring during an infection (though larger sample sizes would be needed to lower the false discovery rate and pinpoint biologically relevant interactions).

10.1128/mSystems.00116-20.9 FIG S1 Examples of high correlations between host and parasite gene expression levels across samples. Each dot shows the expression levels of a selected human (*x* axis) and P. falciparum (*y* axis) gene pair in one blood sample. The coefficient of correlation is indicated above each plot. The four plots represent the correlations between the human genes RECQL4 (RecQ-like helicase 4), CDIPT (CDP-diacylglycerol-inositol 3-phosphatidyltransferase), CFAP45 (cilia- and flagellum-associated protein 45) and FRS3 (fibroblast growth factor receptor substrate 3) and, respectively, the P. falciparum genes PF3D7_0930200 (leucine-rich repeat protein), PF3D7_1460700 (60S ribosomal protein L27), PF3D7_1332400 (nucleotidyltransferase), and PF3D7_0814200 (DNA/RNA-binding protein Alba 1). Download FIG S1, TIF file, 0.6 MB. Copyright © 2020 Bradwell et al. 2020 Bradwell et al. This content is distributed under the terms of the Creative Commons Attribution 4.0 International license.

### Gene expression deconvolution allows determination of the relative proportions of WBC subsets and parasite developmental stages.

**FIG 4:**
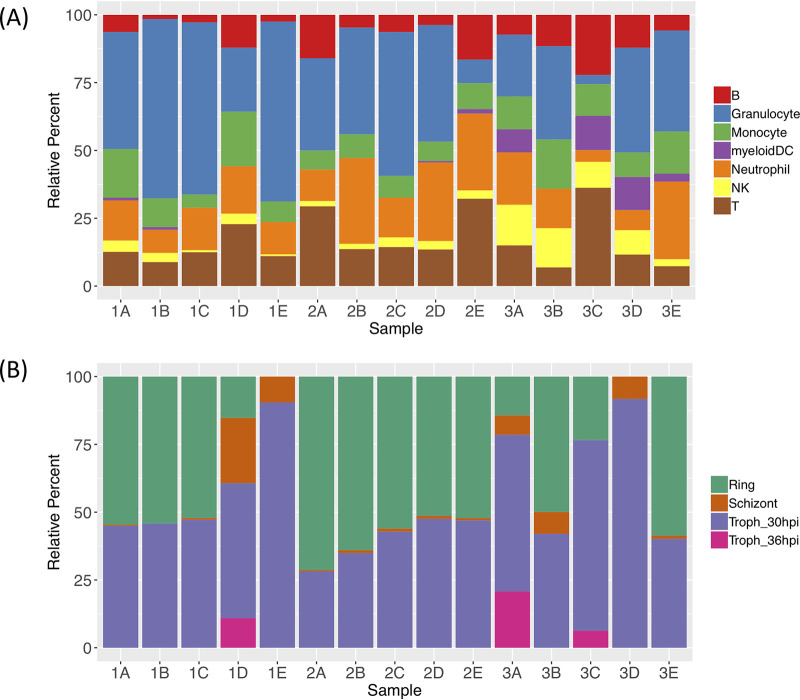
Gene expression deconvolution results. (A) Relative proportions of the different white blood cell subsets determined from the host transcriptomes. (B) Relative proportions of the different P. falciparum developmental stages determined from the parasite transcriptomes (hpi, hours postinfection).

Host gene expression data generated from whole blood can be difficult to interpret as the samples contain a variable proportion of cell types, each with their own specific regulation, and gene expression differences between samples could simply reflect differences in cell composition. Similarly, parasite gene expression profiles will be influenced by the relative proportions of different parasite developmental stages. To overcome these limitations and determine the proportions of WBC subsets and parasite developmental stages in each sample, we used gene expression deconvolution analysis (24). First, we used transcriptome profiles from sorted WBCs (25–28), as well as P. falciparum developmental stage transcriptome profiles obtained from single-cell RNA-seq (29) to generate the gene expression signature profiles of each cell type and parasite stage. We then used these signature profiles to deconvolute the complex gene expression profiles generated from whole blood and statistically separate the transcriptional signal from each cell and parasite stage (Fig. 4).

Overall, the proportions of the different white blood cell subsets inferred from the RNA-seq data matched those expected in human whole blood (30), except for sample 3C, which displayed a low proportion of granulocytes and relatively high proportions of T cells, B cells, and myeloid dendritic cells. Interestingly, the proportion of NK cells seemed to decrease with the infection number (*P* = 2.0 × 10^−3^), though the small proportion of NK cells in each sample warrants caution. Similarly, the proportion of transcripts derived from myeloid dendritic cells and NK cells seemed to differ significantly among individuals (*P* values of 0.03 and 1.8 × 10^−5^, respectively) (Table S6).

10.1128/mSystems.00116-20.6 TABLE S6 Variation in the proportions of white blood cells by successive infection numbers and by patient. Download Table S6, XLSX file, 0.01 MB. Copyright © 2020 Bradwell et al. 2020 Bradwell et al. This content is distributed under the terms of the Creative Commons Attribution 4.0 International license.

In contrast, the proportion from different parasite developmental stages did not seem to change between individuals (*P* > 0.06) or as a function of the number of infections (*P* > 0.11) (Table S7). Note that the small number of samples in the current study prevented us from correcting the differential expression analyses described above for these variations in composition, but larger studies could easily integrate this information to correct for differences among samples and distinguish whether the differential expression is caused by differences in cell composition or genuine differences in specific transcript regulation.

10.1128/mSystems.00116-20.7 TABLE S7 Variation in the proportions of parasite development stages by successive infection numbers and by patient. Download Table S7, XLSX file, 0.01 MB. Copyright © 2020 Bradwell et al. 2020 Bradwell et al. This content is distributed under the terms of the Creative Commons Attribution 4.0 International license.

### Complexity of infection and genotyping.

**FIG 5:**
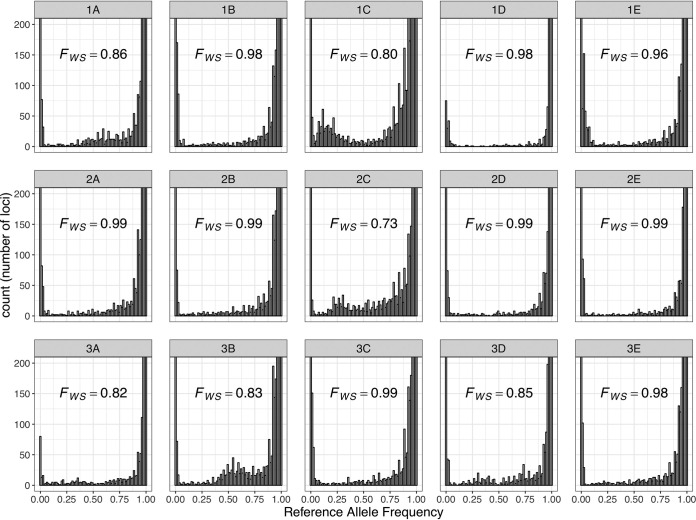
Complexity of infection analysis. The reference allele frequency distributions show, for each sample, the number of nucleotide positions (*y* axis) with a given proportion of reads carrying the reference allele (*x* axis). Note that while most infections show a clear U-shaped distribution consistent with the presence of a single (haploid) clone, infections 1A, 1C, 2C, and 3B display clear multimodal distributions consistent with the presence of multiple, genetically different parasites. The corresponding *Fws* values are indicated in each plot (with *Fws *< 0.95 indicative of polyclonal infections).

In addition to the mixture of parasite stages, *Plasmodium* infections often simultaneously contain multiple, genetically distinct clones. Since *Plasmodium* parasites are haploid in the human host, identification of multiple alleles throughout the genome is indicative of a polyclonal infection. To evaluate whether RNA-seq data distinguishes monoclonal from polyclonal infections, we analyzed allelic variations, within each infection, at nucleotide sites highly sequenced (>50×) using the sequences generated by RNA-seq. While most infections displayed a single allele at each transcribed position, allelic variation patterns in six infections were suggestive of the presence of two or more clones (Fig. 5). These observations were consistent with the *Fws* value (47), an estimate of polyclonality akin to Wright’s inbreeding coefficient and calculated by comparing the heterozygosity within and between infections, determined from each infection: six samples displayed an *Fws *of <0.95, indicative of multiple clones present in these infections (Fig. 5). We could hypothesize that, as the patients acquire immunity over successive P. falciparum infections, they would be infected with fewer clones but we did not observe any association between polyclonality and the number of infections (*P* = 0.19) (nor with the patient identifier [ID], *P* = 0.5), although more samples will be required to rigorously evaluate this hypothesis.

We also used genetic information extracted from the RNA-seq data to examine relationships among the dominant P. falciparum clone of each infection. All clones appeared equally distant from each other (Fig. S2), regardless of whether they were observed in successive infections of the same child or in different children. This analysis is consistent with successful drug treatment following each infection and indicates that consecutive infections in the same individual were caused by new infections rather than by recrudescence of resistant parasites.

10.1128/mSystems.00116-20.10 FIG S2 Neighbor-joining tree showing the genetic relationships among the dominant clone of each infection based on pairwise nucleotide differences. Download FIG S2, TIF file, 0.4 MB. Copyright © 2020 Bradwell et al. 2020 Bradwell et al. This content is distributed under the terms of the Creative Commons Attribution 4.0 International license.

## DISCUSSION

Here, we applied dual RNA-seq to analyze whole-blood samples collected from three Malian children over five successive P. falciparum clinical infections. We successfully obtained more than one million reads from each sample to characterize both host and parasite transcriptomes, allowing robust analysis of differential gene expression, discovery of extensive host and parasite gene coexpression, determination of the proportions of the WBC subsets and parasite developmental stages, assessment of the complexity of infection, and parasite genotyping.

One striking result from this analysis was the different patterns of clustering of the host and parasite transcriptomes generated from the same infections: host gene expression profiles appeared to be quantitatively more affected by the individual than by the number of previous infections, while the parasite transcriptomes tended to separate early from late infections. This pattern, which was observed using unsupervised clustering and gene-by-gene analysis, could indicate that transcriptional changes occur in P. falciparum parasites in order to successfully infect more malaria-experienced hosts (although the number of genes identified in our analyses remained small, and additional samples would be required to rigorously validate this hypothesis). Similarly, and despite the larger quantitative interindividual variations, many host genes were statistically associated with sequential clinical infections and could hint at the molecular mechanisms involved in the acquisition of immunity against falciparum malaria. Thus, we observed differential host expression of several immunoregulatory genes (Fig. 2A), including PILRα and BTN2A2, that were upregulated in successive infections, and DNTTIP2 and MAP3K8, that were downregulated. PILRα is one member of an immunoglobulin-like receptor gene pair and acts as an innate immune system signaling inhibitor (32). BTN2A2 inhibits T-cell metabolism, IL-2 and gamma interferon (IFN-γ) secretion, and CD4 and CD8 T-cell proliferation (33). MAP3K8 induces production of NF-kappa β, a potent inducer of proinflammatory genes (34). These findings are consistent with a progressive dampening of the host inflammatory response over successive infections and mirror some of the gene expression changes described in malaria-experienced hosts compared to malaria-naive hosts (15). Table S5, displaying GSEA results and leading edge genes driving enrichment, shows that within the enriched pathways there are immunoregulatory genes that increase in expression with infection number such as suppressors of cytokine signaling and cytokine-inducible SH2-containing protein, and suppressors of interferon such as interferon regulatory factor 2 (IRF2) which competitively inhibits IRF1-mediated activation of interferons alpha and beta (35). Interleukin 6 receptor and IL-6 signal transducer genes are also present on significantly enriched pathways. Interleukin 6 has both pro- and anti-inflammatory roles, and inhibits the proinflammatory IL-1 as well as activates the anti-inflammatory IL-10, and the latter has previously been suggested to be involved in antidisease immunity to malaria (18). The identification of genes involved in platelet regulation as differentially regulated upon successive infections is interesting, as platelets have been shown to be involved in parasite killing and clumping of P. falciparum-infected erythrocytes, which leads to thrombocytopenia (one complication of malaria) (7). GSEA has previously been used to analyze transcriptional changes during controlled human malaria infection (CHMI), and it is interesting to note the similarities in enriched pathways, including platelet activation and GTPase-mediated signaling found over successive infections in this study compared to days postinoculation versus baseline in P. falciparum CHMI (36), and P. vivax CHMI of naive versus semi-immune individuals (37).

Note here that it is possible that the children had malaria episodes prior to enrollment in our study and that infection 1 does not correspond to the child’s first malaria infection (although given the young age of the children studied, it is probably one of their first). Techniques used herein, such as differential expression, GSEA, gene signature-based deconvolution, and correlation of host and parasite gene expression, have been used elsewhere for human and *Plasmodium* transcriptomic analysis (15, 16, 19, 36, 38). However, as highlighted in a recent review (38), there is extensive variability in the human subjects compared and techniques used to understand development of malaria immunity, a lack of guidance on methodology to aid defining and characterizing naturally acquired immunity, and absence of detailed time course or infection number transcriptional changes within the same individual. In addition, very little is known about parasite adaptations across successively more malaria-experienced hosts. Overall, while the small sample size of the current study prevents drawing definitive conclusions, our study demonstrates that dual RNA-seq over successive infections can provide a solid framework to better understand transcriptional changes in the parasite and the host accompanying the development of acquired immunity in malaria patients.

Beyond testing for gene expression differences, we leveraged the RNA-seq data to determine the relative proportions of WBC subsets and parasite developmental stages in each sample using gene expression deconvolution (24). Our findings demonstrate that whole-blood RNA-seq is not critically hampered by the cell heterogeneity of each sample but, in contrast, can provide important information and facilitate measurement of changes in WBC subsets over time, and if sample size is sufficient, to correct differential gene expression analyses for these changes to distinguish changes in cell proportions from a difference in gene regulation in a specific cell population. However, we noted that, using gene expression deconvolution, it was difficult to accurately differentiate and quantify cell populations that have similar transcriptional profiles. In particular, we were not able to reliably differentiate CD4^+^ and CD8^+^ T-cell subsets in our analyses and, despite their different biological roles, had to combine these two populations into a single category, though recent progress in gene expression deconvolution methods could address this issue (39).

Finally, we show that data generated by RNA-seq enable determination of the complexity of each infection and comparison of the genotype of the clones in different samples. This information is critical for studies of successive infections to ensure that the samples analyzed truly represent new infections and not recrudescence, from previous infections, of parasites that are resistant to antimalarial drugs or have been incompletely cleared. This approach could also allow assessment of the role of polyclonality, and possibly of specific parasite genetic polymorphisms, in the response to successive infections. Note however that the determination of allelic variants from RNA might fail to identify polyclonal infections if the different clones in one infection are present at different developmental stages. If this is the case, analyses of genomic DNA might be necessary to avoid misclassifying possible asynchronous polyclonal infections as monoclonal.

Overall, we show that RNA-seq data generated from whole-blood samples collected from children with malaria can provide a wide variety of information to better understand host and parasite changes accompanying the acquisition of immunity against malaria. In addition to the analysis of differential gene expression of the host and parasite associated with successive clinical infections, our study demonstrates that the RNA-seq data can enable identifying host/pathogen interactions, measuring (and correcting for) the proportion of the white blood cell subsets and parasite developmental stages, and determining the clone genotypes and the number of clones present in each infection. The biological complexity of clinical malaria infections involves interactions between a large number of host, parasite, and environmental factors, which would require analyses on a much larger sample size than presented here. A greater number of samples would, for example, enable a rigorous analysis of the interaction between the sex of the host and gene expression of both the host and parasite across successive infections. While the current study is limited by its sample size, application of the approaches implemented here to a larger cohort could provide a novel and comprehensive perspective on the dynamic changes in host and parasite regulation and their interactions during the acquisition of immunity to the disease and could highlight key molecular processes that could then be leveraged to develop more efficient treatment and prevention approaches against malaria.

## MATERIALS AND METHODS

### Sample collection.

Whole-blood samples were collected from five successive symptomatic, uncomplicated infections in three Malian children aged ∼1 to 2 years using PAXgene blood RNA tubes (PreAnalytiX). The presence of P. falciparum in each sample was confirmed via light microscopic examination of thick blood smears, with no detectable presence of other parasitic species.

### Ethics approval and consent.

The study protocol and informed consent/assent process were reviewed and approved by the institutional review boards of the Faculty of Medicine, Pharmacy and Dentistry of the University of Sciences, Techniques and Technologies of Bamako and the University of Maryland, Baltimore (IRB numbers HCR-HP-00041382 and HP-00085882). Individual written informed consent was obtained from parents or guardians.

### Generation of RNA-seq data.

RNA was extracted from PAXgene tubes using the Blood RNA kit (Qiagen) and used to prepare stranded libraries after rRNA and globin depletion using the TruSeq Stranded RNA kit (Illumina) and poly(A) selection using the TruSeq RNA sample preparation v2 kit (Illumina). cDNA libraries were sequenced on an Illumina HiSeq 4000 to generate paired-end reads of 75 bp. To test whether any infection contains more than one *Plasmodium* species, we first randomly subsampled 2,500,000 reads from each fastq file using seqtk v1.3 (https://github.com/lh3/seqtk) and aligned those reads using hisat2 v2.0.4 (40) to a fasta file containing the P. falciparum 3D7, P. vivax PvP01, P. cynomolgi M version 2, P. knowlesi H strain, *P. malariae* UG01, and *P. ovale* GH01 genomes from PlasmoDB v36 (31). We then counted the number of reads mapped uniquely to each genome using samtools view. We aligned all reads using hisat2 (v2.0.4) (40) to (i) the P. falciparum 3D7 genome (PlasmoDB v36 [31]) (with the default parameters except for --max-intronlen 5000, --score-min L,0,-0.4) and (ii) to nonredundant autosomal sequences from the human hg38 genome. We then filtered out any reads mapping to both genomes (always less than 0.17%) and removed potential PCR duplicates with samtools v1.7 markdup. We calculated read counts per gene using the gene annotations downloaded from PlasmoDB (plasmodb.org, for *Plasmodium* genes) and NCBI (for the human genes) and custom python scripts (available at https://github.com/kbradwell/malaria-dualTranscriptomics).

### Gene expression analysis.

The read counts per gene were normalized into count per million reads mapped separately for the human and parasite genes. Unsupervised clustering was performed after calculating Euclidean distances between transcriptomes using the R functions dist() and hclust() (v3.3.1). Statistical assessment of differential gene expression was performed using EdgeR v3.16.5 (41) using simultaneously the number of successive infections and patient ID as covariates (without interactions) and a quasilikelihood negative binomial generalized model. For these analyses, we considered only genes with >10 counts per million in seven or more samples as expressed and tested a total of 8,896 human genes (out of 17,137 human genes) and 2,822 parasite genes (out of 5,558 parasite genes). Inclusion of parasitemia as a covariate did not notably change the results. All results were corrected for multiple testing by FDR (42).

The PANTHER overrepresentation test (release no. 20190308) was performed using Fisher’s exact test with differentially expressed genes (FDR = 0.1) as the test gene set and all 8,896 expressed genes as the reference gene set. GSEA was performed with the R package fgsea v1.0.2 (43), using genes ranked via multiplication of the log fold change with −log_10_(*P* value), 1,000 permutations, and the reactomePathways() function, which uses NCBI stable ID mappings to pathways, to generate normalized enrichment scores and adjusted *P* values for pathway enrichment.

### Gene coexpression analysis.

To determine the extent of coexpression between host and parasite genes, we measured the Spearman correlation coefficient between each pair of human and P. falciparum genes across all samples using the R function cor.test() with method=spearman. To assess significance of the findings, we determined the number of pairwise correlations with a Spearman’s correlation above different *R*^2^ thresholds when randomizing the host and parasite transcriptomes (i.e., by randomly matching the human gene expression profiles and parasite gene expression profiles) and conducting 500 such random permutations. We then determined the significance of the experimental results by calculating the proportion of random permutations with a greater number of pairwise correlations than the number observed at each *R*^2^ threshold) and calculating the enrichment by comparing the number observed experimentally to the average number obtained across all 500 permutations.

### Gene expression deconvolution.

Reference transcriptome profiles for WBC populations were obtained from FACS-sorted RNA-seq studies (25–28) (Table S8). Reference transcriptome profiles for the different P. falciparum developmental stages were obtained from a single-cell RNA-seq study (29). Sufficient male and female gametocyte data were unavailable, and this stage was thus absent from the analysis. Deconvolution was then performed using CIBERSORT v1.06 (24) as described in reference 44. Associations between the proportions of WBC subsets or the parasite developmental stages and successive infections and the child ID were tested by analysis of variance (ANOVA) using the aov() function in Rstudio (v1.0.136).

10.1128/mSystems.00116-20.8 TABLE S8 Description and accession number of the data sets chosen for determining the signature gene expression profiles of human white blood cell for gene expression deconvolution. Download Table S8, XLSX file, 0.01 MB. Copyright © 2020 Bradwell et al. 2020 Bradwell et al. This content is distributed under the terms of the Creative Commons Attribution 4.0 International license.

### Complexity of infection and genotyping.

Reference allele frequency plots were generated for each sample by measuring the proportion of reads carrying the reference P. falciparum allele at each genomic position sequenced >50×. A subset of 3,411,387 positions covered by >50× in at least two samples was used to determine pairwise differences between the dominant clone of each infection (45), and the resulting distance matrix was used to reconstruct a neighbor-joining tree in MEGA v7170509 (46). *Fws* values were determined by the R package moimix, using curated sites (47) with >50× coverage.

### Data availability.

All scripts used in this study are freely available at https://github.com/kbradwell/malaria-dualTranscriptomics. All sequence data are available through NCBI Sequence Read Archive under BioProject accession no. PRJNA591657.
