# Supplementary figures and images for: Host and Parasite Transcriptomic Changes upon Successive Plasmodium falciparum Infections in Early Childhood
Source: mSystems. 2020 Jul 7;5(4):e00116-20. doi: 10.1128/mSystems.00116-20 (PMC7343306; doi:10.1128/mSystems.00116-20)

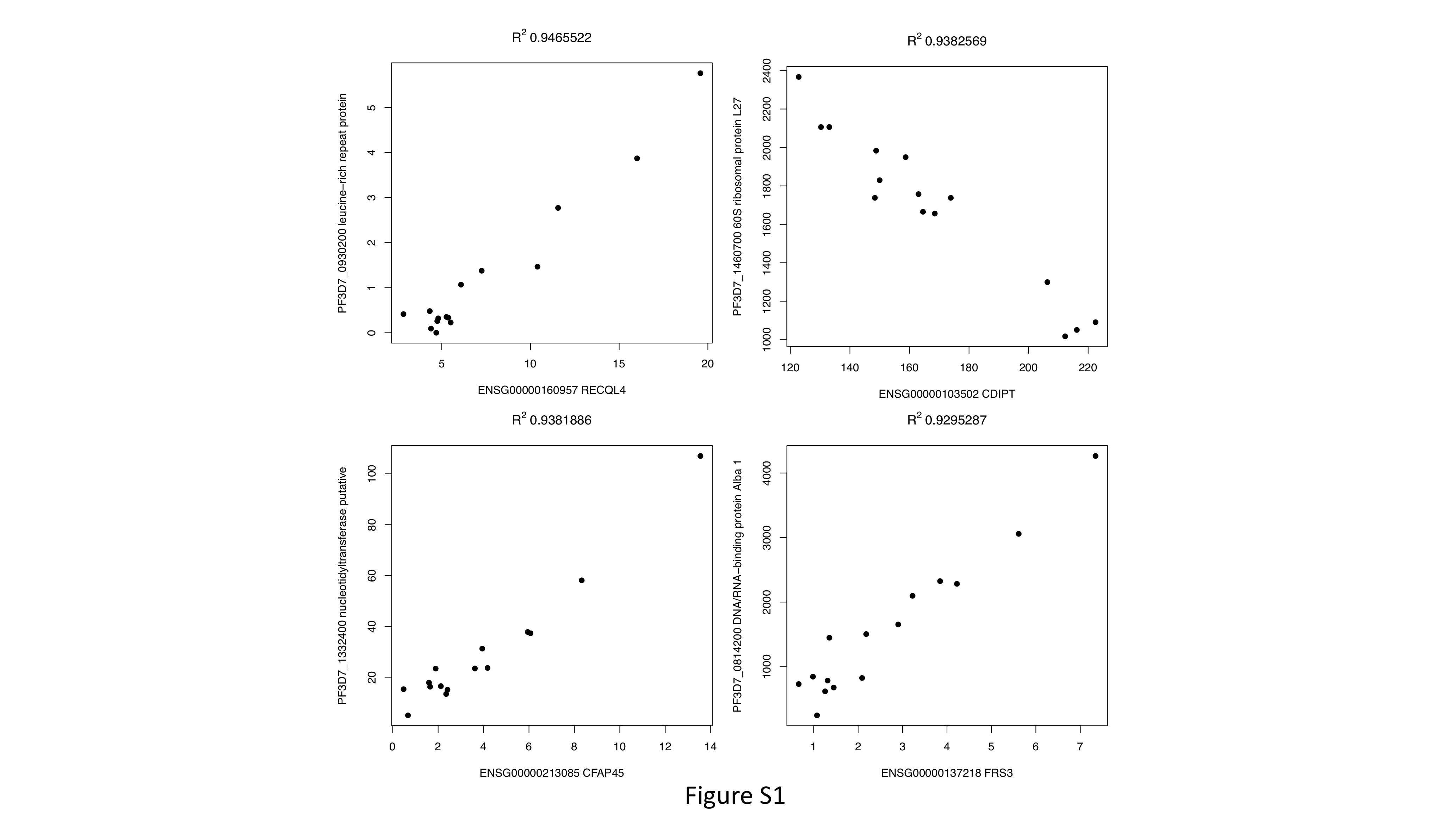

Supplement: FIG S1 [file mSystems.00116-20-sf001.tif]

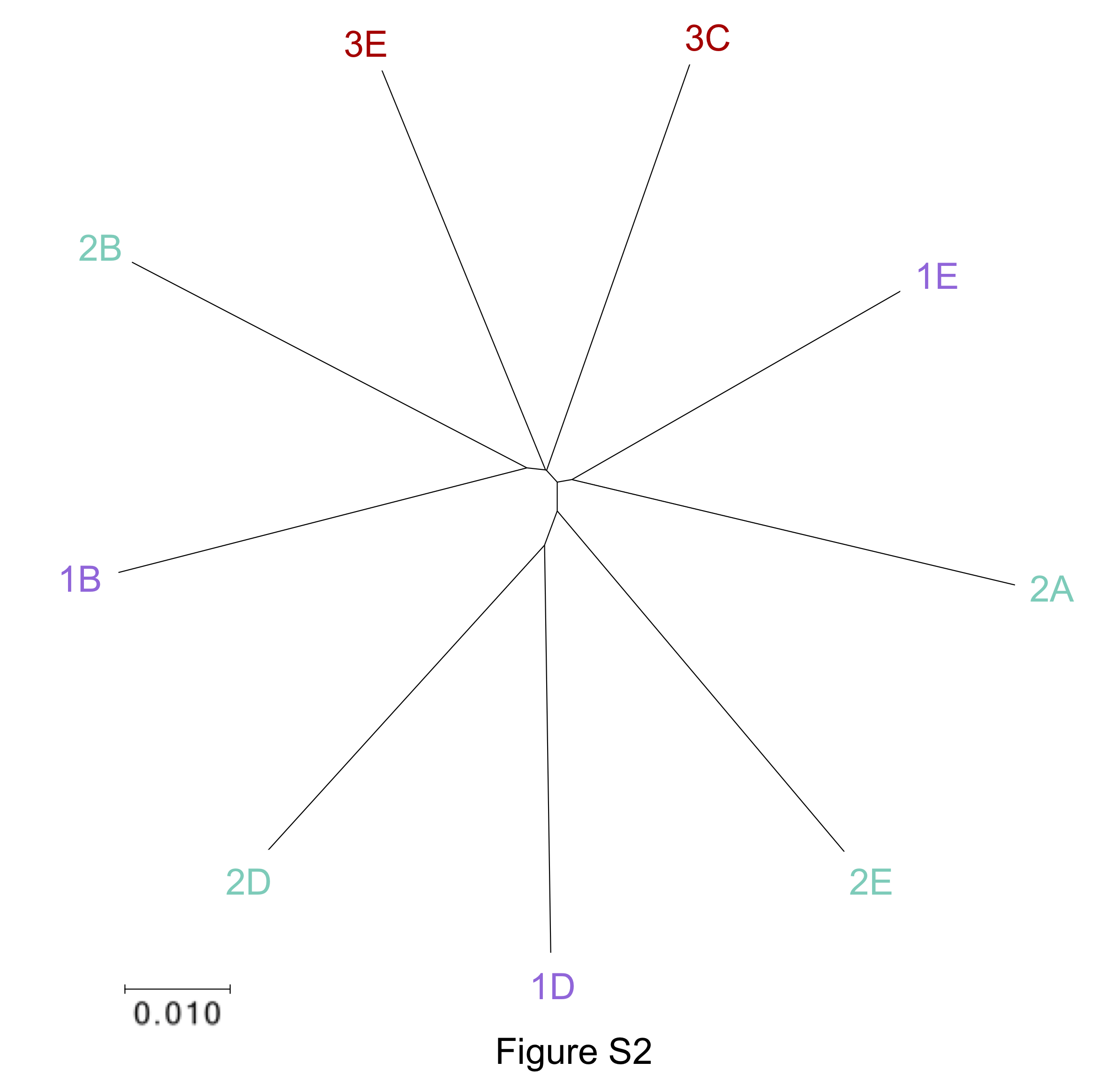

Supplement: FIG S2 [file mSystems.00116-20-sf002.tif]
